# Retraction Note: Long non-coding RNA LINC00968 attenuates drug resistance of breast cancer cells through inhibiting the Wnt2/β-catenin signaling pathway by regulating WNT2

**DOI:** 10.1186/s13046-021-02121-3

**Published:** 2021-10-27

**Authors:** Dian-Hui Xiu, Gui-Feng Liu, Shao-Nan Yu, Long-Yun Li, Guo-Qing Zhao, Lin Liu, Xue-Feng Li

**Affiliations:** 1 Department of Radiology, China-Japan Union Hospital of Jilin University, Changchun, 130033 People’s Republic of China; 2 Department of Anesthesiology, China-Japan Union Hospital of Jilin University, No. 126, Xiantai Street, Changchun, 130033 Jilin Province People’s Republic of China

**Retraction Note: J Exp Clin Cancer Res 38, 94 (2019)**

**https://doi.org/10.1186/s13046-019-1100-8**

The Editor-in-Chief has retracted this article. After publication concerns were raised with respect to the reliability of the western blot data presented. The authors were unable to provide the raw data for this study upon request. The Editor-in-Chief therefore no longer has confidence in the integrity of the data in this article.

Xue-Feng Li has not explicitly stated whether they agree with this retraction. Gui-Feng Liu, Shao-Nan Yu, Long-Yun Li and Lin Liu have not responded to any correspondence from the Editor-in-Chief or Publisher about this retraction. Dian-Hui Xiu and Guo-Qing Zhao agree to this retraction.

